# Estuaries of the Tinto, Odiel and Piedras rivers as source of new species of *Pseudomonas* with biofertilizer potential under stress conditions

**DOI:** 10.1038/s41598-025-31256-y

**Published:** 2025-12-09

**Authors:** Noris J. Flores-Duarte, Ignacio D. Rodríguez-Llorente, Eloisa Pajuelo, Susana Redondo-Gómez, Enrique Mateos-Naranjo, Salvadora Navarro-Torre

**Affiliations:** 1 Departamento de Microbiología y Parasitología, Facultad de Farmacia, Sevilla, Universidad de Sevilla, 41012 Sevilla, Spain; 2 Departamento de Biología Vegetal y Ecología, Facultad de Biología, Sevilla, Universidad de Sevilla, 41012 Sevilla, Spain

**Keywords:** Novel species, PGPB, Sustainable agriculture, Climate change, Legumes, Ecology, Ecology, Environmental sciences, Microbiology, Plant sciences

## Abstract

The estuaries of the Tinto, Odiel, and Piedras rivers have been extensively studied by several groups due to ecological aspects such as heavy metal contamination, nutrient deficiency and degradation influenced by climate change. Aimed to recover these ecosystems, a large collection of plant growth-promoting bacteria (PGPB), several of them previously identified as new species, was isolated from the rhizosphere and tissues of native plants. In this study, we analyse the strains N4, N8^T^, L1^T^, and SDT3^T^ as potential new species. A phylogenomic analysis revealed that four strains belong to the genus *Pseudomonas*, forming distinct clades with full support. Digital DNA-DNA hybridization and average nucleotide identity values were below the species delineation thresholds (70% and 95–96%), confirming the novelty of strains N8^T^, L1^T^, and SDT3^T^, while N4 and N8^T^ were identified as the same species. As a result, we propose the names *Pseudomonas medicaginis* sp. nov., *Pseudomonas onubensis* sp. nov., and *Pseudomonas spartinae* sp. nov., with *P. medicaginis* comprising two strains. These strains, previously identified as PGPB, harboured genes related to PGP traits and were further characterized phenotypically and biochemically. To evaluate the agroecological potential of N8^T^ strain, it was tested in co-inoculation with the corresponding rhizobia on lentil, pea, alfalfa, and bean under greenhouse conditions. In all legumes, N8^T^ enhanced shoot and root biomass, increased nodule numbers, and improved nitrogen content compared with single rhizobia inoculation. In summary, these estuarine ecosystems are not only ecologically significant for their microbial diversity but also serve as reservoirs of beneficial bacteria with promising agroecological applications.

**Supplementary Information:**

The online version contains supplementary material available at 10.1038/s41598-025-31256-y.

## Introduction

Estuaries are complex ecosystems of great ecological importance that are often under a great pressure due to both human activities and environmental changes related to climate change. However, the activities carried out in estuaries constitute an economic support for the communities that inhabit them, which take advantage of the natural resources they offer. The joint estuary of the Tinto and Odiel rivers in Huelva (Southwest Spain) is a widely studied ecosystem considered as one of the most polluted regions of the world^1^. Tinto and Odiel rivers collect the drainage of a metalliferous mining area in the Iberian Pyrite Belt, rich in massive metal sulfide deposits which have been exploited during centuries for the extraction of metals, particularly gold, silver and copper^2^. The estuary area is also affected by the industrial activity in the adjacent areas, such as oil refineries, paper mill, and fertilizer factories, among others^3^. On the other hand, although the Piedras river estuary located close to this ecosystem does not have pollution problems, many of its soils are poor in nutrients, which greatly limits the plant species that can grow in them^4^.

Aimed to recover these ecosystems using autochthonous plants and their associated bacteria, we focused our research on the isolation and characterization of plant growth-promoting bacteria (PGPB), both rhizospheric and endophytic, from the microbiota of halophytes and salt-tolerant legumes that grow in the Tinto-Odiel estuary such as *Spartina densiflora*^5^*, Spartina maritima*^6–8^*, Arthrocnemum macrostachyum*^9^*, Halimione portulacoides* and *Salicornia ramosissima*^10^*,* and *Medicago* spp.^3^. Concerning the estuary of Piedras river, PGPB from the rizosphere of *Medicago* spp. plants growing in nutrient poor soils were isolated and characterized^4^. As a result of these works, we have designed different bacterial synthetic communities (SynComs), most of them including *Pseudomonas* strains, that have demonstrated their utility to promote the growth of several plants of agronomic interest under different stresses. In that way, these SynComs were able to improve tomato plants tolerance against acute heat wave stress^11^, strawberry tolerance against severe drought, soil salinization and short extreme heat event^12,13^, rice response to climate change conditions^14^ or grapevine heat stress resilience^15^, among others. Concerning legumes, a SynCom nodule isolated from *Medicago* spp. was able to improve *Medicago sativa* growth and nodulation under different abiotic stresses^16^. Prospecting the microbiota associated to halophyte plants in the estuaries also resulted in the identification of a good amount of new species of bacteria belonging to *Marinomonas*^17^, *Vibrio*^18^, *Kushneria*^19^, *Halomonas*^20^, *Pseudoalteromonas*^21^ and *Rosellomorea*^22^ genera. These ecosystems have proved to be a source of both biotools with potential as biofertilizers and bacterial species not described to date, whose genomes could be useful to study the evolution of plant-bacteria relationships under stress.

The environmental ubiquity of the *Pseudomonas* genus has been widely described and the number of new species belonging to this genus increases year by year^23^. The purposes of this work were i) to describe three new bacterial species belonging to the genus *Pseudomonas* isolated from the rhizosphere or inside plants growing in the joint estuary of the Tinto and Odiel rivers and the estuary of Piedras river, with the ability to promote plant growth under stress conditions, and ii) to demonstrate the utility of one of them, here described as *Pseudomonas medicaginis* N8^T^, as biofertilizer for grain legumes.

## Material and methods

### 16S rRNA gene phylogeny

From previous works, the bacterial collection of the BIO-181 group contains strains N4, N8^T^, L1^T^ (also called MIS C2^T^) and SDT3^T^ of genus *Pseudomonas* that could be new species based on their genetic characterization. The strains N4 and N8^T^ were isolated from nodules of *Medicago* spp. plants growing in the Odiel marshes (Huelva, Spain)^3^. From the Tinto River estuary (Huelva, Spain), strain SDT3^T^ was isolated from the *Spartina densiflora* rhizosphere^5^. On the other hand, strain L1^T^ was isolated from the rhizosphere of *Medicago* spp. plants growing in the Piedras river estuarine nutrient poor soils (Huelva, Spain)^4^. These strains were deposited in the Spanish Type Culture Collection (CECT) and the Belgian Coordinated Collections of Microorganisms (BCCM) sending a pure culture of each strain according to the depositing instructions for each centre.

In that works, 16S rRNA gene sequence of these strains were deposited in DDBJ/EMBL/GenBank databases under the following accession numbers: OP060610 (N4), OP060614 (N8^T^), OM397092 (L1^T^) and JX047434 (SDT3^T^).

16S rRNA gene sequences were compared with type strains using the EzBioCloud database^24^ and a phylogenetic tree with the closet species was inferred using the GGDC web server^25^.

### Genome sequencing and analysis

The whole genome of the four strains was sequenced. The N4 strain genome was sequenced by the MicrobesNG company (Birmingham, UK) using the hybrid method (combination of Illumina and Oxford Nanopore technologies). On the other hand, the N8^T^, L1^T^ and SDT3^T^ genomes were sequenced using Illumina technology by Sistemas Genómicos (Valencia, Spain) (for N8^T^) and MicrobesNG (for L1^T^ and SDT3^T^). In all cases, the bacterial strains were sent to the respective company, and the company staff isolated the DNA. For the genomes sequenced by MicrobesNG, reads were trimmed using Trimmomatic^26^, and the quality was determined using an in-house script. Assembly was performed using SPAdes version 3.7^27^ for Illumina reads, and Unicycler version 0.4.0^28^) for hybrid method. For the genome sequenced by Sistemas Genómicos, reads were trimmed using an in-house script, the quality was checked using FASTQC^29^, and the assembly was performed with Megahit^30^. All genomes were annotated with Prokka version 1.14.5^31^. Assembly metrics and other basic statistics were obtained using QUAST v. 5.3.0 software^32^, RAST server version 2.0^33^, CRISPRCasFinder^34^, and CheckM v1.2.3^35^.

To create the phylogenomic tree, genomes were analysed using the Type (Strain) Genome Server (TYGS)^36^. Phylogenetic inference was performed using FastME 2.1.4, including SPR postprocessing^37^. The tree was rooted at the midpoint^38^ and visualized with PhyD3^39^.

Finally, to determine the novelty of the species, digital DNA-DNA hybridization (dDDH) and average nucleotide identity (ANI) tests of the study genomes and the closet species were performed using the GGDC web server^25^ and the JSpeciesWS server^40^, respectively.

The whole genomes were deposited in the DDBJ/EMBL/GenBank databases with the following accession numbers: OZ221631 (N4), CBFHBB01 (N8^T^), CBFHAZ01 (L1^T^) and CBDAMB01 (SDT3^T^).

### Physiological and biochemical characterization

The strains were characterized based on their phenotypic features and biochemical properties.

For phenotypic features, strains were grown in TSA plates (Tryptic Soy Agar; Intron Biotechnology) at different temperatures (5, 10, 15, 20, 25, 30, 35, 40 and 45 °C), different NaCl concentrations (0, 0.5, 1, and 1.5 M; at 30 °C) and different pH values (4, 6, 7, 8, 9, 10 and 11; at 30 °C) during 10 days. NaCl concentrations were added to the TSA before autoclaving the correspond amount of a 4 M NaCl stock solution. pH values were adjusted using a citrate–phosphate buffer (0.1 M citric acid and 0.2 M dibasic sodium phosphate) and a Tris–HCl buffer (0.1 M Tris (hydroxymethyl) aminomethane and 0.1 M HCl). After that, the optimal growth conditions were determined.

With optimal growth conditions for each strain, the morphology, size, and colour of the colonies in TSA plates were observed using a stereoscopic microscope (Olympus SZ61), and the size and morphology of the cells were observed using an optical microscope with an objective × 100 (Olympus CX41) after Gram staining^41^. Motility was also verified with the optical microscope with an objective × 40 using a drop of liquid culture in TSB (Tryptic Soy Broth) incubated under optimal growth conditions for each strain.

For biochemical characteristics, an oxidase test was performed adding 1% N,N,N′,N′-tetramethyl-p-phenylenediamine powder (Becton, Dickinson and Company) to the bacterial biomass. Oxidase test was considered positive if the colour of the biomass turned blue. Catalase activity was tested adding a drop of 3% H_2_O_2_ to the bacterial biomass. The presence of bubbles indicated if the test was positive. Finally, an API 20NE gallery (bioMérieux) was performed according to the manufacturer’s instructions for each strain.

### Greenhouse trial with grain legumes

For the greenhouse assay, different legumes species were used: lentil (*Lens culinaris*), pea (*Pisum sativum*), alfalfa (*Medicago sativa*), and bean (*Phaseolus vulgaris*). Seeds of lentil, pea, and alfalfa were purchased from Semillas Batlle S.A. (Barcelona, Spain) and seeds of bean were purchased from Semillas Wam® (Pontevedra, Spain), and stored at 4 °C before use. First of all, seeds were surface disinfected by immersion in 70% ethanol, then in sodium hypochlorite, and finally, seeds were rinsed with sterile distilled water several times. The surface disinfection protocol varied according to the legume species^42–44^. Subsequently, 20 seeds of each species were transferred to 0.9% water-agar plates, and incubated in darkness at 28 °C. Once pre-germination was observed, seedlings were transferred to pots (11 cm × 11 cm × 12 cm) containing a sterilised substrate (coconut fibre : perlite; ratio 3:1). The substrate was sterilized in an autoclave at 121 °C and 1 atm overpressure for 30 min, repeated twice.

Two seedlings were planted per pot, with eight pots per treatment in each legume species. Inoculation treatments were as follows: Rhizobial strain (inoculation with the corresponding rhizobial strain: *Rhizobium leguminosarum* bv. *viciae* ISL10 for lentil and pea plants, *Ensifer* sp. N10 for alfalfa plants, and *Rhizobium tropici* CIAT 899 for bean plants) and rhizobial strain + N8 (co-inoculation with the corresponding rhizobial strain and *Pseudomonas medicaginis* N8^T^ tagged with mCherry). The different bacterial inocula were performed as described Flores-Duarte et al^16^. Briefly, strains were cultivated in liquid medium (TY for rhizobial strains and TSB for strain N8^T^) at 24 h in agitation and then, the pellet for each culture, after a centrifugation, was resuspended in 0.9% sterile saline solution. For co-inoculation treatment the corresponding rhizobium was mixed with N8^T^. Strains used in this work belong to the bacterial collection of the BIO-181 group and strain N8^T^ labelled with mCherry fluorescent protein was performed in a previous work^3^.

Plants were watered weekly with 50 mL of sterile water and inoculated weekly with 50 mL of the corresponding bacterial inoculum. Greenhouse conditions were controlled: natural light was supplemented with artificial lighting to maintain a 16 h light/8 h dark photoperiod, and temperatures were set to 25 °C during the day and 15 °C at night. The experiment lasted 60 days.

At the end of the experiment, plants were recollected, and shoots and roots lengths were measured, and the number of nodules were counted. Then, shoots and roots were separated, dried in an oven at 80 °C for 48 h, and the dry weight was determined.

Finally, nitrogen content in plant tissues was measured using an InfrAlyzer 300 analyzer (Technicon, Tarrytown, NY, USA) on dry material, following the protocol described by Carrasco et al^45^. Macro and micronutrient concentrations were determined by the ionomic service of CEBAS (Murcia, Spain) using inductively coupled plasma optical emission spectrometry (ICP-OES) with an ARL Fisons 3410 instrument (Thermo Scientific, Waltham, MA, USA).

### Statistical analysis

All data were first checked for normal distribution. Statistical comparisons among treatments were performed using ANOVA followed by Fisher’s test, using Statistica version 6.0 software (StatSoft Inc., Tulsa, OK, USA).

## Results and discussion

### Description of a new species of the genus *Pseudomonas*

Bacterial strains were identified as *Pseudomonas* based on the 16S rRNA gene sequence, and the closest species were *Pseudomonas mucoides* P154a^T^ with percentages of identity of 97.47% (for strain N4) and 99.13% (for strain N8^T^), *Pseudomonas edaphica* RD25^T^ with a 99.92% (for strain L1^T^) and *Pseudomonas composti* CCUG 59231^ T^ with a 99.59% of identity (for strain SDT3^T^). The phylogenetic tree showed the same results except for strains N4 and N8^T^ which appeared in the same clade with *Pseudomonas citri* OPS13-3^ T^ as the closest species (identity percentage of 97.30% and 98.5%, respectively) (Supplementary Fig. 1). These results suggest that the four strains could be new species of the *Pseudomonas* genus. It can be observed that in the case of strains N4 and N8^T^ the closest species changes between the sequence comparison and the phylogenetic tree and it is due to fundamental differences in both methods to assess similarity and evolutionary relationships. It should be noted out that even an identity over 99% could identify a new species, as strains L1^T^ and SDT3^T^ showed. For that reason, we suggest that the identification of bacterial strains based on 16S rRNA gene sequence should be at genus level to avoid wrong identifications in the databases and the literature. As described below, to assign a strain to a species, genome comparison should be performed.

### Genome analysis

The whole genome of the four strains was sequenced, and the features are represented in Supplementary Table 1.

**Fig. 1:**
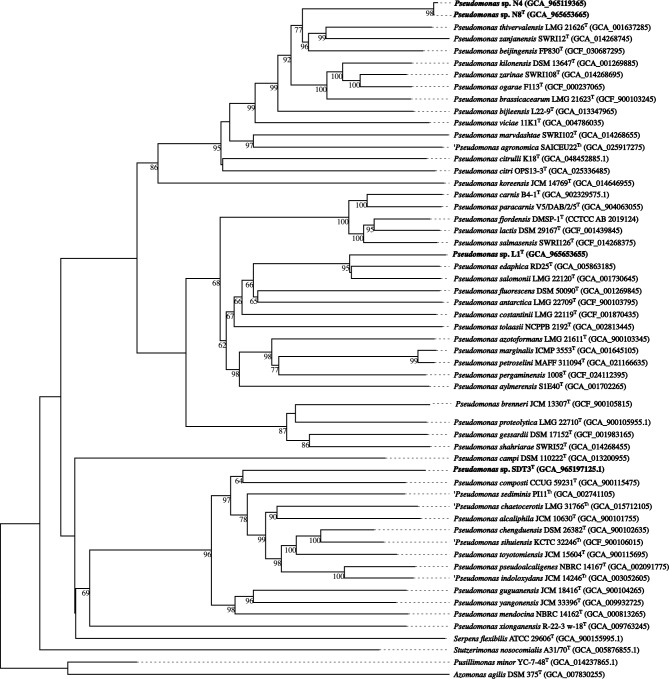
Phylogenomic tree inferred from GBDP distances calculated from genome sequences showing the phylogenetic position of strains N4, N8^T^, SDT3^T^ and L1^T^ relative to closest species. the numbers above branches are GBDP pseudo-bootstrap support values from 100 replications with an average branch support of 96.8%. Assembly accession numbers are given in parentheses.

Phylogenomic trees were constructed (with the genomes) in order to determinate more accurate the phylogenetic position of the four strains. Regarding strains N4 and N8^T^, Fig. 1 showed that both strains belong to the same clade of *Pseudomonas thivervalensis* LMG 21626^ T^*, Pseudomonas zanjanensis* SWRI12^T^ and *Pseudomonas beijingensis* FP830^T^. The overall genome related indexes (OGRI) calculated comparing these two strains with the closely related species showed that both strains belong to the same species because dDDH value was higher than 70% and ANIs values were higher than 95–96%^46^. With the same criteria, both strains belong to a new species because the OGRI values were lower than these thresholds in the closely related species, being *P. beijingensis* FP830^T^ the closest one (Table 1). In the case of strain L1^T^, it shared the clade with *Pseudomonas salomonii* LMG 22120^ T^ and *P. edaphica* RD25^T^ (Fig. 1), and OGRI results confirmed that strain L1^T^ is a new species of the genus *Pseudomonas* (Table 1). Finally, phylogenomic tree of SDT3^T^ matched with the phylogenetic tree based on 16S rRNA gene being *P. composti* CCUG 59231^ T^ the closest one with a dDDH value of 38.90% and ANI values of 88.80% and 90.21% (ANIb and ANIm, respectively) (Fig. 1 and Table 1). These results confirmed that strain SDT3^T^ is also a new specie of the genus *Pseudomonas*.

**Table 1:** OGRI results of strains N4, N8^T^, L1^T^, and SDT3^T^.

	dDDH					
*Pseudomonas* sp. N8^T^	*Pseudomonas* sp. N4	*P. zanjanensis* SWRI12^T^	*P. beijingensis* FP830^T^	*P. thivervalensis* LMG 21626^ T^	*P. citri* OPS13-3^ T^	*P. mucoides* P154a^T^
	99.10%	44.60%	47.40%	47.00%	34.30%	26.70%
	**ANIb**					
	99.83%	90.95%	91.75%	91.17%	86.68%	81.29%
	**ANIm**					
	99.89%	92.27%	92.80%	92.60%	88.84%	86.09%
*Pseudomonas* sp. L1^T^	*P. salomonii* LMG 22120^ T^			*P. edaphica* RD25^T^		
	**dDDH**					
	56.80%			56.70%		
	**ANIb**					
	93.35%			93.43%		
	**ANIm**					
	94.95%			94.68%		
*Pseudomonas* sp. SDT3^T^	*P. composti* CCUG 59231^ T^					
	**dDDH**					
	38.90%					
	**ANIb**					
	88.80%					
	**ANIm**					
	90.21%					

Mining the whole genome of the four strains, important genes were founded such as genes related to fatty acids, quinones, polar lipids, and PGP properties (Supplementary Tables 2 and 3). Biosynthesis of fatty acids in bacteria occurs through the FASII system^47^. Strains N4, N8^T^, and SDT3^T^ had all the genes implicated in the FASII system, but strain L1^T^ had not completed this pathway due the absence of the *fabB* and *hadA* genes which are responsible for the initial condensation of malonyl-ACP and the dehydration of intermediates, respectively. However, L1^T^ had a long-chain-fatty acid ligase (*fadD1* gene) whose substrates come from different pathways and they use them to synthetize fatty acids (Supplementary Table 2)^48^.

Regarding respiratory quinones, the major one is Q9 in genus *Pseudomonas* as well in the closest species of the four strains, *P. edaphica*, *P. citri* and *P. beijingensis*^49–51^. In the genome of strain SDT3^T^, all the genes related to the biosynthesis of quinone Q9 were found (Supplementary Table 2). However, some genes such us *ubiI* in strains N4 and N8^T^, and *ubiD* in L1^T^ were not found (Supplementary Table 2), suggesting that these strains have alternative enzymes to synthetize the quinone Q9. To finish with the chemotaxonomic analyses by searching the genes in the genomes, strains N4, N8^T^ and SDT3^T^ presented genes to synthetize diphosphatidylglycerol (DPG), phosphatidylglycerol (PG), phosphatidylethanolamine (PE), phosphatidylserine (PS) and phosphatidylcholine (PC) as polar lipids, while strain L1^T^ had the genes for the biosynthesis of DPG, PE, PS and PC (Supplementary Table 2). Although polar lipids are not studied in all the closest species, these results are in consonance with those obtained for *P. citri* and *P. beijingensis*^50,51^.

On the other hand, since these strains were selected for their ability to promote plant growth^3–5^, genes related to PGP properties were also searched (Supplementary Table 3). Regarding auxin production, none of the four strains described in this study presented a tryptophan-dependent pathway for IAA synthesis, although strains N4, N8^T^ and L1^T^ were positive for indole-3-acetic acid (IAA) in previous works^3–5^. The IAA synthesis by these strains could be achieved by a tryptophan-independent pathway as happens in other microorganisms^52–54^. Strains N4 and N8^T^ also showed ACC deaminase activity and the gene *acdS* was found in their genomes (Supplementary Table 3). Four strains were able to solubilize phosphates and showed genes related to, such as phosphatases, in their genomes (Supplementary Table 3). The ability to produce siderophores was also present in the four strains, showing several genes related to this activity in their genomes, such as pyoverdine biosynthesis genes, bacillibactin and enterobactin exporters, achromobactin, ferrichrome and ferric-anguibactin receptors, etc. (Supplementary Table 3). Pyoverdine is a typical fluorescent siderophore described in several species of the genus *Pseudomonas*^55^. Related to biofilm formation, the *pga* and *psl* operons were found in the described strains and, in addition, different genes for flagellum formation, indicating that four strains are motiles (Supplementary Table 3) as typically described in *Pseudomonas*. Finally, despite the possible ability to fix nitrogen described for strains N4, N8^T^, and L1^T^^3,4^, *nifH* gene was not found in their genomes. As explained in previous works, the fact that strains grew in a minimal medium without a nitrogen source is not an irrefutable proof to affirm that bacteria are nitrogen fixers, due to the possible presence of nitrogen traces. The presence of all these genes related to PGP properties supports the effect of these strains on plant growth promotion under stress conditions in different experiments *in planta*. For example, bacterial SynComs including strain SDT3^T^ enhanced the growth of different crops under abiotic stresses related to the climate change such as heat waves, drought, salinity and high CO_2_ concentration^11–15^. On the other hand, strains N4 and N8^T^ improved the growth and the nodulation of *M. sativa* under heavy metal pollution, drought, salinity and high temperature, demonstrating the capacity to be used both as biorfetilizer and to phytostabilizate polluted soils with heavy metals^16^. Concerning L1^T^, in single inoculation or being part of a SynCom, it showed positive effects at growth and nodulation levels in alfalfa under nutrient deficiency^4^. Currently, these strains have been included in several SynComs and formulations are being developed for their use as biofertilizers.

### Physiological and biochemical characteristics

To study the physiological characteristics of the four strains, the optimal growth conditions were first determined. Strains N4, N8^T^ and L1^T^ grew from 5 °C to 40 °C while the maximum tolerable temperature for SDT3^T^ was 40 °C and the minimum 10 °C. Regarding the NaCl concentration, the maximum tolerable NaCl concentration was 1 M in the case of strain N4, and 0.5 M for N8^T^, L1^T^ and SDT3^T^. All of them were able to grow in the absence of NaCl, confirming they are halotolerant bacteria. All strains grew at a pH value of 6 to 9, but SDT3^T^ also grew at a pH value of 10. After observation of bacterial growth under the different conditions, the optimal condition was in TSA plates (pH = 7) at 30 °C for 24 h for all strains. When comparing each strain with its closely type specie, it can be observed in Table 2 that the strains described in this study tolerated higher temperatures than the related species, but in general, the conditions for growth were very similar among them.

**Table 2:** Physiological and biochemical features of studied strains and the most related species.

	*Pseudomonas* sp. N4	*Pseudomonas* sp. N8^T^	*Pseudomonas* sp. L1^T^	*Pseudomonas* sp. SDT3^T^	*P. beijingensis* FP830^T^	*P. salomonii* LMG 22120^ T^	*P. edaphica* RD25^T^	*P. composti* CCUG 59231^ T^
Temperature range (°C)	5–40	5–40	5–40	10–40	4–37	n.d.−37	n.d.−36	4–37
NaCl range (M)	0–1	0–0.5	0–0.5	0–0.5	0–0.7	n.d	0–1	0–1.1
pH range	6–9	6–9	6–9	6–10	6–9	n.d	5–9	n.d
Nitrate reduction	+	+	–	–	+	–	–	–
Indole production	–	–	–	–	–	+	–	–
**Enzymatic activities:**								
Urease	–	+/–	–	+	–	n.d	+	–
Arginine dihydrolase	+	+	+	+	–	+	+	–
Esculin hydrolase	–	–	–	–	+	–	–	–
Gelatinase	–	–	–	–	–	+	–	–
**Assimilation of:**								
L-arabinose	+	+	+	–	+	+	+	+
D-mannose	–	+	+	+	+	n.d	+	+
D-mannitol	+	+	+	+	–	n.d	+	+
N-acetyl-glucosamine	–	–	+	–	+	–	+	–
D-maltose	–	–	–	–	–	n.d	+	–
Malic acid	+	+	+	+	+	+	–	+
Potassium gluconate	–	–	–	–	+	n.d	+	+
Phenylacetic acid	+	+	–	+	–	n.d	–	–

Data of the studied strains was obtained in this work. Data of *P. beijingensis* FP830^T^, *P. salomonii* LMG 22120^ T^, *P. edaphica* RD25^T^, and *P. composti* CCUG 59231^ T^ was obtained from^49,51,57,58^, respectively.

+ : positive; –: negative; +/–: weak; n.d.: no determinate.

Under these optimal conditions, the aspect of the colonies for each strain was detailed. N4 and N8^T^ showed the same aspect, small light-yellow colonies of 4 mm in diameter, convex, irregular, with undulate edges and a smooth and wet aspect. When the culture grew older, the colour of the colonies changed to dark brown. L1^T^ colonies were light yellow with a 3 mm in diameter, convex, irregular with undulate edges and a smooth and wet aspect. Finally, colonies of strain SDT3^T^ showed a size of 3.5 mm in diameter with a filamentous edge, flat, smooth and wet appearance with a mustard yellow colour. Cells from these colonies were individual Gram-negative rods of 1 µm × 1.6 µm (N4), 1 µm × 2.2 µm (N8^T^), 1 µm × 2 µm (L1^T^), and 1 µm × 3 µm (SDT3^T^), and motiles. These results are in line with other species of *Pseudomonas*^56^.

To end with the bacterial description, the biochemical features were studied (Table 2). All strains were positive for oxidase and catalase as all *Pseudomonas* species^49,51,56–58^. API 20NE test results revealed that the four strains had strong activity of the arginine dihydrolase, and were able to assimilate D-glucose, D-mannose, potassium gluconate, capric acid, D-maltose, and citric acid. On the other hand, only strain L1^T^ was able to assimilate N-acetyl-glucosamine. Nitrate to nitrogen (N_2_) reduction was performed only by strains N4 and N8^T^, and urease activity was observed in SDT3^T^ and N8^T^, but very weakly in the last strain. Finally, the assimilation of L-arabinose was observed in N4, N8^T^, and L1^T^, malic acid was assimilated by strains N8^T^, L1^T^, and SDT3^T^ while phenylacetic acid was assimilated by N4, N8^T^, and SDT3^T^. Although strains N4 and N8^T^ belong to the same specie according to the genomic approximation, there were some differences in biochemical features such as urease activity and D-mannose assimilation (Table 2). In this line, both strains showed differences in the presence of arginine dihydrolase and esculin hydrolase, and the assimilation of N-acetyl-glucosamine, D-mannose, potassium gluconate, and phenylacetic acid with *P. beijingensis* FP830^T^^51^. The few differences between the suggested new species and the most related ones also happened in strain L1^T^, which was negative for the indole production, urease and gelatinase activities, and D-maltose and potassium gluconate assimilation, and positive for N-acetyl-glucosamine and malic acid assimilation while *P. salomonii* LMG 22120^ T^ and *P. edaphica* RD25^T^ showed contrary results^49,57^. In addition, strain SDT3^T^ also showed different results with the related species *P. composti* CCUG 59231^ T^ in the urease and arginine dihydrolase activities and in the assimilation of L-arabinose, potassium gluconate, and phenylacetic acid^58^. The phenotypic and biochemical similitudes of different species of genus *Pseudomonas* and these few differences with the closest species support the genomic results confirming that the four strains are new species of the genus *Pseudomonas* being strains N4 and N8^T^ the same species. The proposed names for N8^T^, L1^T^ and SDT3^T^ are *Pseudomonas medicaginis* sp. nov., *Pseudomonas onubensis* sp. nov., and *Pseudomonas spartinae* sp. nov., respectively.

## Strain N8^T^ boosts growth across grain legumes: a promising biofertilizer

Due to previous results obtained using strain N8^T^ to inoculate the forage legume alfalfa under different stressful conditions^3,16^, we tested in this study the beneficial effect of this strain in different species of grain legumes to describe its potential as biofertilizer for legume crops.

### Plant growth improvement

**Fig. 2:**
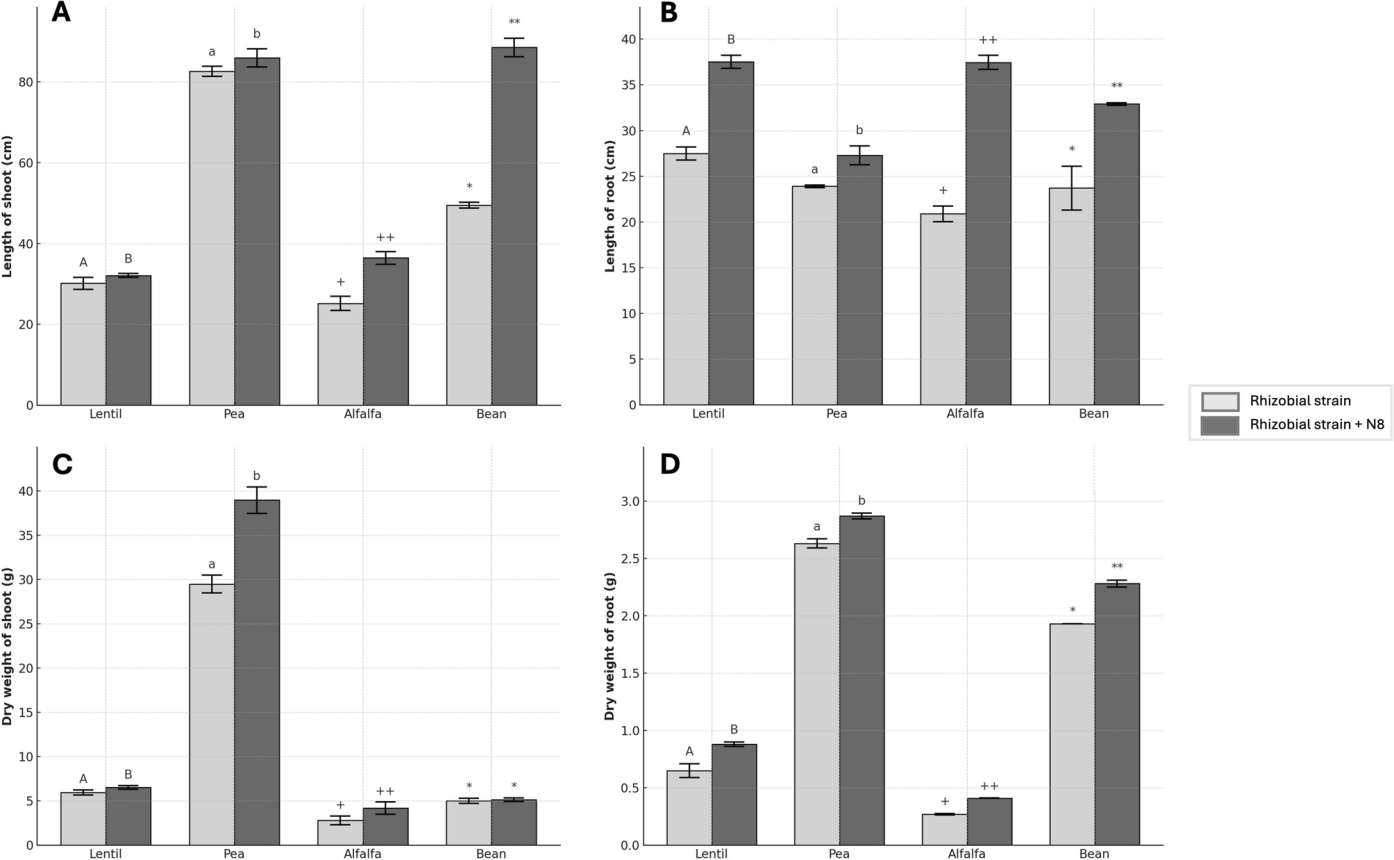
Effects on plant growth of different leguminous plants inoculated with strain N8^T^ and different rhizobia. lengths of shoots (**A**) and roots (**B**), and dry weight of shoots (**C**) and roots (**D**) after 60 days sowing in pots with perlite and commercial substrate. values represent the mean ± standard deviation (n = 16). different letters and symbols indicate statistically significant differences between means. lowercase and uppercase letters and the symbols “ + ”, and “*” are used to distinguish different plant species and should not be compared to each other (one-way ANOVA, LSD test,* p* < 0.001). the different treatments were rhizobial strain (inoculation with the corresponding rhizobial strain: *Rhizobium leguminosarum* bv. *viciae* ISL10 for lentil and pea plants, *Ensifer* sp. N10 for alfalfa plants, and *Rhizobium tropici* CIAT 899 for bean plants) and rhizobial strain + N8 (co-inoculation with the corresponding rhizobial strain and *Pseudomonas medicaginis* N8^T^ tagged with mCherry).

Four legumes (lentil, pea, alfalfa, and common bean) were inoculated with strain N8^T^ and the corresponding rhizobia in order to observe the effect of N8^T^ in plant growth and nodule development. Co-inoculation significantly stimulated shoot and root elongation (Fig. 2AB). The most substantial effects were observed in alfalfa co-inoculated with *Ensifer* sp. N10 + *P. medicaginis* N8^T^, and in bean with *Rhizobium tropici* CIAT 899 + *P. medicaginis* N8^T^. In both species, the longest roots were recorded following co-inoculation, while lentil and pea also showed significant differences. Bean exhibited the highest shoot length (reaching up to 90 cm) and the greatest relative increase in roots (~ 35 cm to ~ 42 cm). These effects could be linked to the production of growth-stimulating compounds by *Pseudomonas*, such as siderophores and ACC deaminase, which reduce ethylene stress and promote cell elongation^16,59^ and to the ability of certain endophytes to modulate root architecture, enabling access to deeper substrate layers^16,59,60^ Additionally, the stimulated root development promotes greater water and nutrient uptake, contributing integrally to plant growth and vigor^61^. Regarding dry weight of shoots (Fig. 2C), a significant increase was observed in lentil, pea and alfalfa following co-inoculation compared to inoculation with the rhizobia alone. This effect was more pronounced in pea, reaching values of approximately 40 g. On the other hand, the dry weights of roots were improved in all co-inoculated plants with statistical differences (Fig. 2D). These results suggest a synergistic effect of the endophyte on biomass accumulation and are consistent with previous studies indicating that *Pseudomonas* spp. can promote plant growth and lateral root development through the production of phytohormones such as auxins and cytokinins^3,59,61,62^.

### Nodule development and nitrogen content

**Fig. 3:**
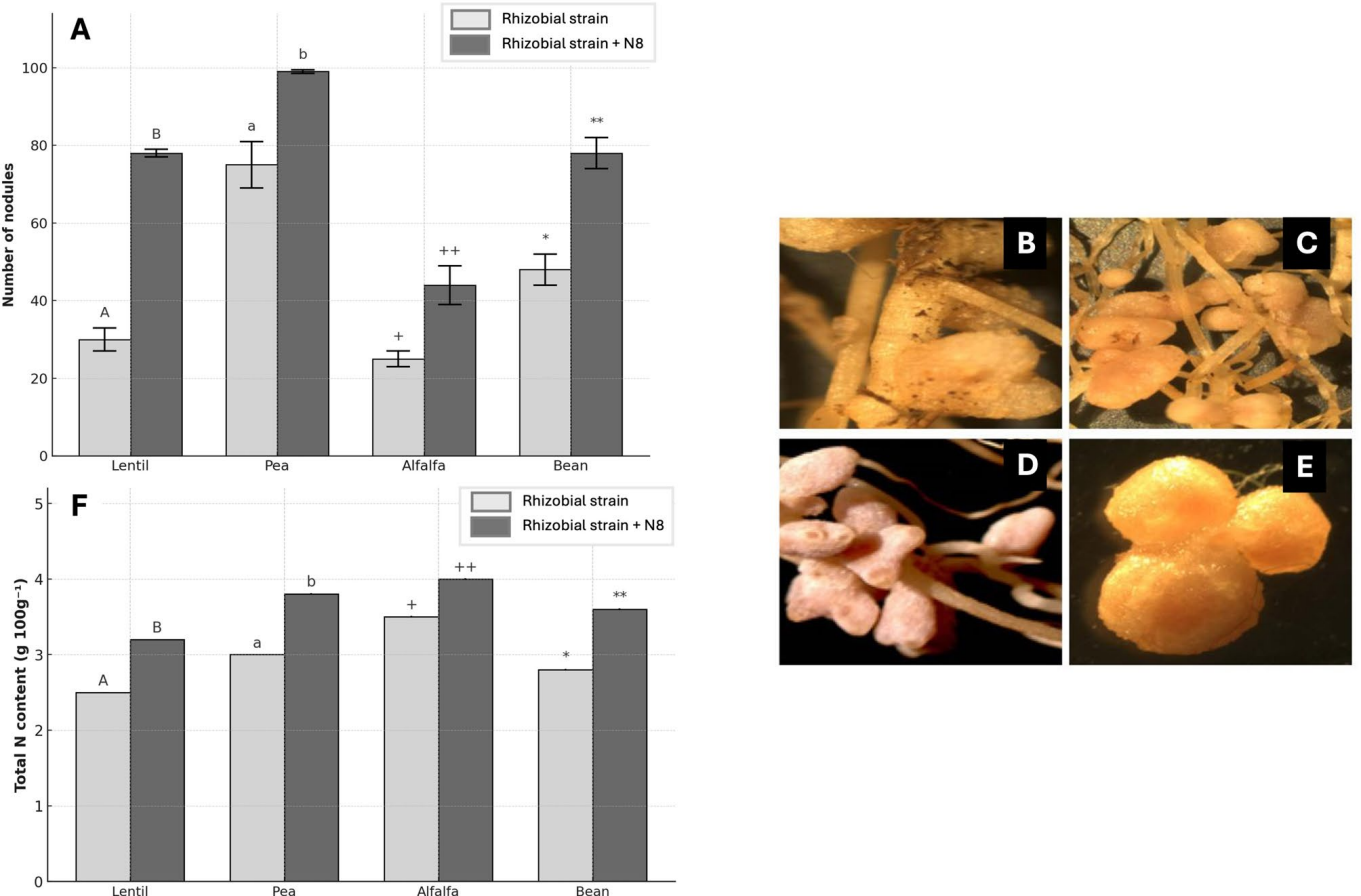
Effects on the nodulation and the total nitrogen content of different leguminous plants inoculated with strain N8^T^ and different rhizobia. Number of nodules (**A**), nodules morphology of co-inoculated plants of lentil (**B**), pea (**C**), alfalfa (**D**), and bean (**E**), and total nitrogen content (**F**) after 60 days sowing in pots with perlite and commercial substrate. values represent the mean ± standard deviation (n = 16). different letters and symbols indicate statistically significant differences between means. lowercase and uppercase letters and the symbols “ + ”, and “*” are used to distinguish different plant species and should not be compared to each other (one-way ANOVA, LSD test,* p* < 0.001). the different treatments were rhizobial strain (inoculation with the corresponding rhizobial strain: *Rhizobium leguminosarum* bv. *viciae* ISL10 for lentil and pea plants, *Ensifer* sp. N10 for alfalfa plants, and *Rhizobium tropici* CIAT 899 for bean plants) and rhizobial strain + N8 (co-inoculation with the corresponding rhizobial strain and *Pseudomonas medicaginis* N8^T^ tagged with mCherry).

In addition to the plant growth, the effect in the nodule development was also observed. Results indicated that co-inoculation significantly increased the number of nodules in all species compared to single inoculation with rhizobia (Fig. 3A). Although pea exhibited the highest number of nodules (~ 100 nodules) under co-inoculated conditions, the highest increase was observed in lentil, approximately a 166%, followed by alfalfa and bean (80% and 60%, respectively). Although pea showed the lowest improvement in the number of nodules, it also responded positively to the co-inoculation treatment. Several mechanisms may explain this effect. For instance, the production of auxins by *P. medicaginis* N8^T^ could promote root hair curling and cortical cell division, facilitating nodule initiation^3,63^. Improved root architecture may also create more infection sites for rhizobia^59^. Endophytes like *Pseudomonas* can modulate plant defense responses, reducing inhibition of nodule formation in leguminous plants, thus increasing number of nodules^59,64^. Previous studies have reported similar synergistic effects by strain N8^T^ in alfalfa under different abiotic stress^16^ and by *Pseudomonas* sp. MRS13 in *Cicer arietinum* (chickpea)^65^. Furthermore, it has been shown that ACC deaminase activity and IAA synthesis play a significant role in the nodulation process, as they help delay nodule senescence, promote interaction with the bacteroid and the activation of genes related to the legume–rhizobium symbiosis^63^.

Regarding the morphology of nodules formed under co-inoculation treatments, they were well-developed, reflecting efficient infection and active nodule organogenesis (Fig. 3). Interestingly, all these nodules were bigger in size than those formed under single rhizobia inoculation. In lentil, pea and alfalfa indeterminate type nodules were observed (Fig. 3BCD), while bean nodules appeared large and spherical features (Fig. 3E).

Related to these results, the total nitrogen content in plants was determined, being significantly higher in co-inoculated legumes (Fig. 3F). Lentil and bean showed the most notable increases (28% and 27%, respectively). In contrast, alfalfa exhibited the lowest increase in nitrogen content (14%). The fact that lentil showed the highest increase in nitrogen content is consistent with the number of nodules, but the result obtained in alfalfa could suggest that, although alfalfa showed a high increase in the number of nodules, they could be less efficient. The contrary could be observed in pea plants. The increase in nitrogen levels could be attributed to more efficient biological nitrogen fixation, likely due to enhanced nodule size and functionality^66^, the endophyte’s contribution to root health and nutrient uptake^67^, or the possible upregulation of *nif/fix* genes in rhizobia when co-inoculated with PGPB^68^.

### Endophytic behaviour by strain N8^T^

The localization of the strain *P. medicaginis* N8^T^ tagged with the fluorescent protein mCherry in nodules was observed (Supplementary Fig. 2). The red fluorescence observed in the images indicates the presence of labelled bacteria in the nodular zones, particularly in the nitrogen fixation region. The fact that strain N8^T^ acts as endophyte in nodules of alfalfa was demonstrated previously by Flores-Duarte et al^3^, but in this work we demonstrated that strain N8^T^ also colonizes nodules of other grain legumes.

### Macro and micronutrients accumulation in plant tissues

Additionally, macro and micronutrients content were determined in each legume in order to measure the capability of N8^T^ to promote nutrient uptake and accumulation in plant tissues (Table 3). Co-inoculation showed a significant effect on mineral uptake, particularly in roots, where substantial increases in iron, zinc, and potassium were recorded. In contrast, the effects observed in shoots were more attenuated or even negative for certain elements. Lentil plants showed an increase in all the measured nutrients in both shoots and roots, and alfalfa showed lower amount of most nutrients in co-inoculated plants than in single inoculated, both in shoots and roots. Similar results were observed in shoot of pea plants. The highest increase in nutrients content was observed in co-inoculated bean plants, increasing a 459% of iron in roots. These findings are consistent with recent studies reporting that endophytes such as *Pseudomonas* spp. facilitate the solubilization and mobilization of key nutrients for plant development such as iron through siderophores production and zinc, thus promoting their accumulation in root tissues and subsequent translocation^69,70^. Similarly, other recent studies have reported enhanced nodulation and improved absorption of nutrients such as Fe and Zn following co-inoculation in various legume species^3,16,71^. The marked increase in root mineral accumulation suggests that co-inoculation induces a root effect that enhances plant nutrition, stimulates biological nitrogen fixation, and could potentially contribute to crop biofortification.

**Table 3:** Accumulation of macro and micronutrients in tissues of different leguminous plants after 60 days sowing in pots with perlite and commercial substrate.

	Ca (g/100 g)	Fe (mg/Kg)	K (g/100 g)	Mg (g/100 g)	Mn (mg/Kg)	Na (g/100 g)	P (g/100 g)	Zn (mg/Kg)
**Shoots**								
Single inoculation- lentil	1.2564±0.01^A^	64.4044±0.57^A^	2.8670±0.00^A^	0.2875±0.00^A^	68.0032±0.00^A^	0.0825±0.00^A^	0.4153±0.04^A^	43.1067±0.15^A^
Co-inoculation- lentil	1.5584±0.02^B^	71.9888±0.01^B^	3.8663±0.00^B^	0.3460±0.00^B^	74.3611±0.51^B^	0.1522±0.00^B^	0.5365±0.00^B^	56.1497±0.21^B^
Single inoculation - pea	1.8353±0.01^a^	83.4605±0.65^a^	4.1382±0.19^a^	0.3733±0.00^a^	83.0027±0.00^a^	0.0334±0.00^a^	0.6393±0.01^a^	57.4686±0.66^a^
Co-inoculation- pea	1.4781±0.03^b^	66.9987±0.00^b^	4.1711±0.24^b^	0.2666±0.00^b^	50.6198±0.53^b^	0.0432±0.00^b^	0.5213±0.00^b^	55.5176±0.68^b^
Single inoculation - alfalfa	1.6284±0.03^+^	73.4951±0.70^+^	4.0417±0.05^+^	0.2966±0.00^+^	66.2184±0.30^+^	0.1443±0.00^+^	0.5906±0.01^+^	41.0503±0.07^+^
Co-inoculation- alfalfa	1.4346±0.00^++^	66.7243±0.38^++^	3.9011±0.13^++^	0.2675±0.00^++^	59.9542±0.06^++^	0.1076±0.01^++^	0.5488±0.02^++^	37.7882±0.29^++^
Single inoculation -bean	1.5523±0.00^*^	47.7437±0.36^*^	0.6674±0.00^*^	0.2990±0.00^*^	81.6612±0.47^*^	0.0103±0.01^*^	0.0941±0.01^*^	31.2081±0.29^*^
Co-inoculation-bean	2.0409±0.06^**^	83.9585±0.05^**^	1.1747±0.00^**^	0.4203±0.00^**^	80.7318±0.37^**^	0.0219±0.02^**^	0.0846±0.00^**^	70.5733±0.60^**^
**Roots**								
Single inoculation - lentil	0.8248±0.00^A^	227.8327±0.23^A^	2.8095±0.02^A^	0.2491±0.01^A^	126.9209±0.11^A^	0.2966±0.04^A^	0.5839±0.02^A^	71.9683±0.04^A^
Co-inoculation- lentil	1.1086±0.05^B^	254.1619±1.16^B^	3.3845±0.07^B^	0.3003±0.01^B^	138.5681±0.04^B^	0.3684±0.02^B^	0.7193±0.00^B^	86.8324±0.23^B^
Single inoculation - pea	0.7248±0.02^a^	267.9593±0.05^a^	2.8871±0.04^a^	0.2655±0.08^a^	56.8878±0.15^a^	0.1985±0.02^a^	0.3593±0.02^a^	44.6723±0.46^a^
Co-inoculation- pea	0.7438±0.03^b^	680.7327±0.37^b^	3.0474±0.03^b^	0.2791±0.01^b^	56.9785±0.03^b^	0.2434±0.02^b^	0.3765±0.01^b^	78.5192±0.68^b^
Single inoculation - alfalfa	0.2968±0.00^+^	253.7375±0.37^+^	1.3594±0.01^+^	0.7874±0.04^+^	102.5447±0.64^+^	0.4513±0.04^+^	0.1265±0.01^+^	175.8224±0.25^+^
Co-inoculation- alfalfa	0.6044±0.00^++^	174.5537±0.63^++^	2.8352±0.07^++^	0.2074±0.01^++^	54.7795±0.31^++^	0.1068±0.02^++^	0.7536±0.02^++^	112.5969±0.57^++^
Single inoculation - bean	1.3066±0.01^*^	80.5900±0.57^*^	0.5139±0.02^*^	0.9014±0.01^*^	104.9802±0.03^*^	0.3846±0.00^*^	0.1094±0.01^*^	76.8569±0.20^*^
Co-inoculation- bean	1.2974±0.01^**^	447.7934±0.29^**^	0.9567±0.05^**^	0.8246±0.08^**^	96.9265±0.10^**^	0.2881±0.01^**^	0.0890±0.02^**^	180.7143±0.44^**^

Values are means ± S.D. (n=16). Different letters and symbols indicate statistically significant differences between means. Lowercase and uppercase letters and the symbols “+”, and “*” are used to distinguish different plant species and should not be compared to each other (one-way ANOVA, LSD test, p < 0.001). Single inoculation (inoculation with the corresponding rhizobial strain: *Rhizobium leguminosarum *bv. *viciae *ISL10 for lentil and pea plants, *Ensifer* sp. N10 for alfalfa plants, and *Rhizobium tropici* CIAT 899 for bean plants) and co-inoculation (co-inoculation with the corresponding rhizobial strain and *Pseudomonas medicaginis* N8^T^ tagged with mCherry).

Synergistic interactions between rhizobia and endophytes have been widely reported, particularly when endophytes facilitate nodule colonization, lateral root formation, or tolerance to abiotic stress^66,72^. However, the magnitude of the response varied among species. These differences may be explained by rhizobium–host compatibility, the degree of colonization by *Pseudomonas*, species-specific physiological and root architectural traits, or preferences for specific microbial metabolites or molecular signals^73^.

## Conclusion

The bacterial population associated with the vegetation in the estuaries of the Tinto, Odiel and Piedras rivers have been studied and described by BIO-181 group for a decade. Among all these bacteria, several novel species were found and described. In this way, in this study we describe the potential new species in this collection. Phylogenetic, genomic, phenotypic, and biochemical results confirm that strains N8^T^, L1^T^, and SDT3^T^ are new species of the genus *Pseudomonas,* and strains N4 and N8^T^ are different strains for the same species. These plant-associated bacteria showed PGP properties and some of them demonstrated the ability to promote plant growth in different plant species and under different environmental conditions. In fact, the beneficial effect of strain N8^T^ on plant growth and nodulation of both grain and forage legumes, forming determinate or indeterminate type nodules, is demonstrated in this study.

All these results indicate the ecological importance of the estuaries of the Tinto, Odiel and Piedras rivers at the diversity level and as a source of new species of bacteria with agroecological interest.

### Description of *Pseudomonas medicaginis* sp. nov

*Pseudomonas medicaginis* (me.di.ca’gi.nis. N.L. gen. fem. n. *medicaginis*, from nodules of plant belonging to the genus *Medicago*).

Cells are individual and motile Gram-negative rods. Colonies are light-yellow, convex, irregular, smooth, and wet with undulate edges on TSA plates (pH = 7) at 30 °C for 24 h (optimal conditions). Growth ranges are pH 6–9, 5–40 °C and 0–0.5 M NaCl.

Catalase and oxidase positive. According to API 20NE test, it can reduce nitrate to nitrogen (N_2_), and assimilate D-glucose, L-arabinose, D-mannose, D-mannitol, potassium gluconate, capric acid, malic acid, trisodium citrate, and phenylacetic acid. Indole formation, D-glucose fermentation, aesculin and gelatine hydrolysis, and the assimilation of N-acetyl-glucosamine, D-maltose, and adipic acid is negative. Strong enzymatic activity of arginine dihydrolase, weak activity of urease, and non-activity of β-galactosidase is observed.

The genome of the type strain N8^T^ has 7,074,109 bp assembled in 736 contigs with a mean coverage of 143.9 × and an N50 value of 16,191 bp. The G + C content is 60.8%.

The type strain is N8^T^ (= CECT 30720^ T^ = LMG 32851^ T^) and was isolated from nodules of *Medicago* spp. plants. The GenBank accession number for the 16S rRNA gene sequence is OP060614. The GenBank/EMBL/DDBJ accession number for the draft genome is CBFHBB01.

### Description of *Pseudomonas onubensis* sp. nov

*Pseudomonas onubensis* (o.nu.ben’sis. L. fem. adj. *onubensis*, of or belonging to Onuba, the ancient Latin name of Huelva, a city in Spain, where the type strain was isolated).

Cells are individual and motile Gram-negative rods. Colonies are light-yellow, convex, irregular, smooth, and wet with undulate edges on TSA plates (pH = 7) at 30 °C for 24 h (optimal conditions). The growth ranges are pH 6–9, 5–40 °C and 0–0.5 M NaCl.

Catalase and oxidase positive. According to API 20NE test, it can assimilate D-glucose, L-arabinose, D-mannose, D-mannitol, N-acetyl-glucosamine, potassium gluconate, capric acid, malic acid, and trisodium citrate. Nitrate reduction, indole formation, D-glucose fermentation, aesculin, and gelatine hydrolysis, and assimilation of D-maltose, adipic acid, and phenylacetic acid is negative. Strong enzymatic activity of arginine dihydrolase, and non-activity of urease and β-galactosidase is observed.

The genome of the type strain L1^T^ has 6,653,686 bp assembled in 383 contigs with a mean coverage of 121.3 × and a value of N50 of 50,308 bp. The G + C content is 59.9%.

The type strain is L1^T^ (= MIS C2^T^ = CECT 31157^ T^ = LMG 34132^ T^) and was isolated from the rhizosphere of *Medicago* spp. plants. The GenBank accession number for the 16S rRNA gene sequence is OM397092. The GenBank/EMBL/DDBJ accession number for the draft genome is CBFHAZ01.

### Description of *Pseudomonas spartinae* sp. nov

*Pseudomonas spartinae* (spar.ti’nae. N.L. gen. fem. n. *spartinae*, of *Spartina* the plant genus from which the strain was isolated).

Cells are individual and motile Gram-negative rods. The colonies are yellow mustard, flat, filamentous, smooth and wet with filamentous edges on TSA plates (pH = 7) at 30 °C for 24 h (optimal conditions). The growth ranges are pH 6–10, 10–40 °C and 0–0.5 M NaCl.

Catalase and oxidase positive. According to API 20NE test, it can assimilate D-glucose, D-mannose, D-mannitol, potassium gluconate, capric acid, malic acid, trisodium citrate and phenylacetic acid. Nitrate reduction, indole formation, D-glucose fermentation, aesculin and gelatine hydrolysis, and assimilation of L-arabinose, N-acetyl-glucosamine, D-maltose, and adipic acid is negative. Strong enzymatic activity of arginine dihydrolase and urease, and non-activity of β-galactosidase is observed. The genome of the type strain SDT3^T^ has 5,476,350 bp assembled in 55 contigs with a mean coverage of 95.8 × and an N50 value of 332,243 bp. The G + C content is 62.9%.

The type strain is SDT3^T^ (= CECT 31003^ T^ = LMG 34080^ T^) and was isolated from the rhizosphere of *Spartina densiflora* plants. The GenBank accession number for the 16S rRNA gene sequence is JX047434. The GenBank/EMBL/DDBJ accession number for the draft genome is CBDAMB01.

## Supplementary Information

Supplementary Information.

## Data Availability

16S rRNA gene sequence of the strains were deposited in DDBJ/EMBL/GenBank databases under the following accession numbers: OP060610 (N4), OP060614 (N8 T), OM397092 (L1 T) and JX047434 (SDT3 T). The whole genomes were deposited in the DDBJ/EMBL/GenBank databases with the following accession numbers: OZ221631 (N4; https://www.ncbi.nlm.nih.gov/datasets/genome/GCF_965119365.1/), CBFHBB01 (N8 T; https://www.ncbi.nlm.nih.gov/datasets/genome/GCF_965653665.1/), CBFHAZ01 (L1 T; https://www.ncbi.nlm.nih.gov/datasets/genome/GCF_965653655.1/) and CBDAMB01 (SDT3 T; https://www.ncbi.nlm.nih.gov/datasets/genome/GCA_965197125.1/).
