# Correction: Estuaries of the Tinto, Odiel and Piedras rivers as source of new species of *Pseudomonas* with biofertilizer potential under stress conditions

**DOI:** 10.1038/s41598-026-40408-7

**Published:** 2026-02-17

**Authors:** Noris J. Flores-Duarte, Ignacio D. Rodríguez-Llorente, Eloisa Pajuelo, Susana Redondo-Gómez, Enrique Mateos-Naranjo, Salvadora Navarro-Torre

**Affiliations:** 1 Departamento de Microbiología y Parasitología, Facultad de Farmacia, Sevilla, Universidad de Sevilla, 41012 Sevilla, Spain; 2 Departamento de Biología Vegetal y Ecología, Facultad de Biología, Sevilla, Universidad de Sevilla, 41012 Sevilla, Spain

Correction to: *Scientific Reports* 10.1038/s41598-025-31256-y, published online 09 December 2025

The original version of this Article contained a repeated nomenclatural error where the species name ‘*Pseudomonas medicaginis* sp. nov.’ was incorrectly given as ‘*Pseudomonas medicae* sp. nov*.*’

Consequently, in the Abstract,

“As a result, we propose the names *Pseudomonas medicae* sp. nov., *Pseudomonas onubensis* sp. nov., and *Pseudomonas spartinae* sp. nov., with *P. medicae* comprising two strains.”

now reads:

“As a result, we propose the names *Pseudomonas medicaginis* sp. nov., *Pseudomonas onubensis* sp. nov., and *Pseudomonas spartinae* sp. nov., with *P. medicaginis* comprising two strains.”

In the last paragraph of the Introduction,

“The purposes of this work were i) to describe three new bacterial species belonging to the genus *Pseudomonas* isolated from the rhizosphere or inside plants growing in the joint estuary of the Tinto and Odiel rivers and the estuary of Piedras river, with the ability to promote plant growth under stress conditions, and ii) to demonstrate the utility of one of them, here described as *Pseudomonas medicae* N8^T^, as biofertilizer for grain legumes.”

now reads:

“The purposes of this work were i) to describe three new bacterial species belonging to the genus *Pseudomonas* isolated from the rhizosphere or inside plants growing in the joint estuary of the Tinto and Odiel rivers and the estuary of Piedras river, with the ability to promote plant growth under stress conditions, and ii) to demonstrate the utility of one of them, here described as *Pseudomonas medicaginis* N8^T^, as biofertilizer for grain legumes.”

In the Materials and methods, under the subheading ‘Greenhouse trial with grain legumes’,

“Two seedlings were planted per pot, with eight pots per treatment in each legume species. Inoculation treatments were as follows: Rhizobial strain (inoculation with the corresponding rhizobial strain: *Rhizobium leguminosarum* bv. *viciae* ISL10 for lentil and pea plants, *Ensifer* sp. N10 for alfalfa plants, and *Rhizobium tropici* CIAT 899 for bean plants) and rhizobial strain + N8 (co-inoculation with the corresponding rhizobial strain and *Pseudomonas medicae* N8^T^ tagged with mCherry).”

now reads:

“Two seedlings were planted per pot, with eight pots per treatment in each legume species. Inoculation treatments were as follows: Rhizobial strain (inoculation with the corresponding rhizobial strain: *Rhizobium leguminosarum* bv. *viciae* ISL10 for lentil and pea plants, *Ensifer* sp. N10 for alfalfa plants, and *Rhizobium tropici* CIAT 899 for bean plants) and rhizobial strain + N8 (co-inoculation with the corresponding rhizobial strain and *Pseudomonas medicaginis* N8^T^ tagged with mCherry).”

In the Results and discussion, under the subheading ‘Physiological and biochemical characteristics’,

“The proposed names for N8^T^, L1^T^ and SDT3^T^ are *Pseudomonas medicae* sp. nov.*, Pseudomonas onubensis* sp. nov., and *Pseudomonas spartinae* sp. nov., respectively.”

now reads:

“The proposed names for N8^T^, L1^T^ and SDT3^T^ are *Pseudomonas medicaginis* sp. nov.*, Pseudomonas onubensis* sp. nov., and *Pseudomonas spartinae* sp. nov., respectively.”

In the Strain N8^T^ boosts growth across grain legumes: a promising biofertilizer section, under the subheading ‘Plant growth improvement’,

“The most substantial effects were observed in alfalfa co-inoculated with *Ensifer* sp. N10 + *P. medicae* sp. N8^T^, and in bean with *Rhizobium tropici* CIAT 899 + *P. medicae* N8^T^.”

now reads:

“The most substantial effects were observed in alfalfa co-inoculated with *Ensifer* sp. N10 + *P. medicaginis* sp. N8^T^, and in bean with *Rhizobium tropici* CIAT 899 + *P. medicaginis* N8^T^.”

In the same section, under the subheading ‘Nodule development and nitrogen content’,

“For instance, the production of auxins by *P. medicae* N8^T^ could promote root hair curling and cortical cell division, facilitating nodule initiation^3,63^.”

now reads:

“For instance, the production of auxins by *P. medicaginis* N8^T^ could promote root hair curling and cortical cell division, facilitating nodule initiation^3,63^.”

And, under the subheading ‘Endophytic behaviour by strain N8^T^’,

“The localization of the strain *P. medicae* N8^T^ tagged with the fluorescent protein mCherry in nodules was observed (Supplementary Fig. 2).”

now reads:

“The localization of the strain *P. medicaginis* N8^T^ tagged with the fluorescent protein mCherry in nodules was observed (Supplementary Fig. 2).”

Additionally, in the Figure legends of Figures 2 and 3,

“The different treatments were rhizobial strain (inoculation with the corresponding rhizobial strain: *Rhizobium leguminosarum* bv. *viciae* ISL10 for lentil and pea plants, *Ensifer* sp. N10 for alfalfa plants, and *Rhizobium tropici* CIAT 899 for bean plants) and rhizobial strain + N8 (co-inoculation with the corresponding rhizobial strain and *Pseudomonas medicae* N8^T^ tagged with mCherry).”

now reads:

“The different treatments were rhizobial strain (inoculation with the corresponding rhizobial strain: *Rhizobium leguminosarum* bv. *viciae* ISL10 for lentil and pea plants, *Ensifer* sp. N10 for alfalfa plants, and *Rhizobium tropici* CIAT 899 for bean plants) and rhizobial strain + N8 (co-inoculation with the corresponding rhizobial strain and *Pseudomonas medicaginis* N8^T^ tagged with mCherry).”

In the Table legend of Table 3,

“Single inoculation (inoculation with the corresponding rhizobial strain: *Rhizobium leguminosarum* bv. *viciae* ISL10 for lentil and pea plants, *Ensifer* sp. N10 for alfalfa plants, and *Rhizobium tropici* CIAT 899 for bean plants) and co-inoculation (co-inoculation with the corresponding rhizobial strain and *Pseudomonas medicae* N8^T^ tagged with mCherry).”

now reads:

“Single inoculation (inoculation with the corresponding rhizobial strain: *Rhizobium leguminosarum* bv. *viciae* ISL10 for lentil and pea plants, *Ensifer* sp. N10 for alfalfa plants, and *Rhizobium tropici* CIAT 899 for bean plants) and co-inoculation (co-inoculation with the corresponding rhizobial strain and *Pseudomonas medicaginis* N8^T^ tagged with mCherry).”

In the Conclusion, the protologue,

“*Pseudomonas medicae* (me.di.cae. N.L. gen. fem. n. *medicae*, from nodules of plant belonging to the genus *Medicago*).

Cells are individual and motile Gram-negative rods. Colonies are light-yellow, convex, irregular, smooth, and wet with undulate edges on TSA plates (pH = 7) at 30 °C for 24 h (optimal conditions). Growth ranges are pH 6–9, 5–40 °C and 0–0.5 M NaCl.

Catalase and oxidase positive. According to API 20NE test, it can reduce nitrate to nitrogen (N_2_), and assimilate D-glucose, L-arabinose, D-mannose, D-mannitol, potassium gluconate, capric acid, malic acid, trisodium citrate, and phenylacetic acid. Indole formation, D-glucose fermentation, aesculin and gelatine hydrolysis, and the assimilation of N-acetyl-glucosamine, D-maltose, and adipic acid is negative. Strong enzymatic activity of arginine dihydrolase, weak activity of urease, and non-activity of b-galactosidase is observed.

The genome of the type strain N8^T^ has 7,074,109 bp assembled in 736 contigs with a mean coverage of 143.9× and an N50 value of 16,191 bp. The G+C content is 60.8%.

The type strain is N8^T^ (= CECT 30720^T^ = LMG 30720^T^) and was isolated from nodules of *Medicago spp.* plants. The GenBank accession number for the 16S rRNA gene sequence is OP060614. The GenBank/EMBL/DDBJ accession number for the draft genome is CBFHBB01.”

now reads:

*“Pseudomonas medicaginis* (me.di.ca’gi.nis. N.L. gen. fem. n. *medicaginis*, from nodules of plant belonging to the genus *Medicago*).

Cells are individual and motile Gram-negative rods. Colonies are light-yellow, convex, irregular, smooth, and wet with undulate edges on TSA plates (pH = 7) at 30 °C for 24 h (optimal conditions). Growth ranges are pH 6–9, 5–40 °C and 0–0.5 M NaCl.

Catalase and oxidase positive. According to API 20NE test, it can reduce nitrate to nitrogen (N_2_), and assimilate D-glucose, L-arabinose, D-mannose, D-mannitol, potassium gluconate, capric acid, malic acid, trisodium citrate, and phenylacetic acid. Indole formation, D-glucose fermentation, aesculin and gelatine hydrolysis, and the assimilation of N-acetyl-glucosamine, D-maltose, and adipic acid is negative. Strong enzymatic activity of arginine dihydrolase, weak activity of urease, and non-activity of b-galactosidase is observed.

The genome of the type strain N8^T^ has 7,074,109 bp assembled in 736 contigs with a mean coverage of 143.9× and an N50 value of 16,191 bp. The G+C content is 60.8%

The type strain is N8^T^ (= CECT 30720^T^ = LMG 32851^T^) and was isolated from nodules of *Medicago* spp. plants. The GenBank accession number for the 16S rRNA gene sequence is OP060614. The GenBank/EMBL/DDBJ accession number for the draft genome is CBFHBB01.”

Finally, in the Figure legend of Supplementary Figure 2,

**“Supplementary Fig. 2. Colonization of** ***Pseudomonas medicae*** **N8**^**T**^** in the different legume species.**

Images of colonized nodules of co-inoculated plants with *Pseudomonas medicae* N8^T^ tagged with mCherry of lentil (A), pea (B), alfalfa (C), and bean (D) after 60 days sowing in pots with perlite and commercial substrate.”

now reads:

**“Supplementary Fig. 2. Colonization of** ***Pseudomonas medicaginis*** **N8**^**T**^** in the different legume species.**

Images of colonized nodules of co-inoculated plants with *Pseudomonas medicaginis* N8^T^ tagged with mCherry of lentil (A), pea (B), alfalfa (C), and bean (D) after 60 days sowing in pots with perlite and commercial substrate.”

The original Supplementary Information file is provided below.

The original Article and accompanying Supplementary Information file have been corrected.

## Supplementary Information

Supplementary Information.

