# Supplementary material for: Estuaries of the Tinto, Odiel and Piedras rivers as source of new species of Pseudomonas with biofertilizer potential under stress conditions
Source: Sci Rep. 2025 Dec 9;16:1666. doi: 10.1038/s41598-025-31256-y (PMC12800022; doi:10.1038/s41598-025-31256-y)
Supplement: Supplementary file 1 — Supplementary Information. [file 41598_2025_31256_MOESM1_ESM.docx]

**Supplementary Information**

**Estuaries of the Tinto, Odiel and Piedras rivers as source of new species of *Pseudomonas* with biofertilizer potential under stress conditions**

Noris J. Flores-Duarte^1^, Ignacio D. Rodríguez-Llorente^1*^, Eloisa Pajuelo^1^, Susana Redondo-Gómez^2^, Enrique Mateos-Naranjo^2^, Salvadora Navarro-Torre^1*^

^1^Universidad de Sevilla, Departamento de Microbiología y Parasitología, Facultad de Farmacia, Sevilla, 41012 Sevilla, Spain.

^2^Universidad de Sevilla, Departamento de Biología Vegetal y Ecología, Facultad de Biología, Sevilla, 41012, Sevilla, Spain.

***Correspondence:**[**irodri@us.es**](mailto:irodri@us.es)**;** [**snavarro1@us.es**](mailto:snavarro1@us.es)

**Supplementary Fig. 1.** Maximum-likelihood phylogenetic tree inferred from 16S rRNA gene sequences, showing the phylogenetic position of strains N4, N8^T^, SDT3^T^ and L1^T^ relative to type strains of species within the genus *Pseudomonas*. The branches are scaled in terms of the expected number of substitutions per site. Support values obtained from 1000 replicates from maximum-likelihood (left) and maximum-parsimony (right) bootstrapping are shown above the branches if ≥60 %. Sequence accession numbers are given in parentheses.

| **Supplementary Table 1.** Genomes features | | | | | |
| --- | --- | --- | --- | --- | --- |
| **Feature** | **N4** | **N8^T^** | **SDT3^T^** | **L1^T^** |  |
| Assembly size (bp)^a^ | 7,118,414 | 7,074,109 | 5,476,350 | 6,653,686 |  |
| DNA G+C (%)^a^ | 60.7 | 60.8 | 62.9 | 59.9 |  |
| Number of contigs^a^ | 1 | 736 | 55 | 383 |  |
| Largest contigs (pb)^b^ | 677,411 | 133,156 | 833,557 | 199,324 |  |
| Number of CDS^c^ | 6,150 | 6,166 | 5,026 | 5,820 |  |
| RNA genes^c^ | 69 | 55 | 71 | 68 |  |
| tRNA^c^ | 62 | 52 | 65 | 63 |  |
| rRNA^c^ | 7 | 3 | 6 | 5 |  |
| N50 (pb)^a^ | 7,118,414 | 16,191 | 332,243 | 50,308 |  |
| L50^a^ | 1 | 115 | 5 | 40 |  |
| Mean coverage | 149.8× | 143.9× | 95.8× | 121.3× |  |
| CRISPR reapeats^e^ | 5 | 4 | 0 | 3 |  |

|  |
| --- |
|  |

| ^a^Data from RAST server  ^b^Data from QUAST v. 5.3.0 software  ^c^Data from Prokka v.1.14.5  ^e^Data from CRISPRCasFinder | |
| --- | --- |
|  | |

| **Supplementary Table 2.** Genes related to chemotaxonomy. | | | |  |
| --- | --- | --- | --- | --- |
|  | ***Pseudomonas* sp. N4** | ***Pseudomonas* sp. N8^T^** | ***Pseudomonas* sp. L1^T^** | ***Pseudomonas* sp. SDT3^T^** |
| **Fatty acids** | **FAS-II system:**  Malonyl CoA-acyl carrier protein transacylase (*fadD*), long-chain-fatty-acid--CoA ligase (*fadD1*), acyl carrier protein (*acpM*), acyl carrier protein phosphodiesterase (*acpH*), enoyl-(acyl-carrier-protein) reductase (*inhA*), 3-hydroxyacyl-(acyl-carrier-protein) dehydratase (*hadA*), acetyl-coenzyme A carboxyl transferase α chain (*accD6*), acetyl-coenzyme A carboxyl transferase β chain (*accD6*), biotin carboxylase of acetyl-CoA carboxylase (*accA3*), biotin carboxyl carrier protein of acetyl-CoA carboxylase, 3-oxoacyl-(acyl-carrier-protein) synthase (*fabB*), trans-2-decenoyl-(acyl-carrier-protein) isomerase (*fabM*). | **FAS-II system:**  Malonyl CoA-acyl carrier protein transacylase (*fadD*), acyl carrier protein (*acpM*), long-chain-fatty-acid--CoA ligase (*fadD1*), acyl carrier protein phosphodiesterase (*acpH*), enoyl-(acyl-carrier-protein) reductase (*inhA*), 3-hydroxyacyl-(acyl-carrier-protein) dehydratase (*hadA*), acetyl-coenzyme A carboxyl transferase α chain (*accD6*), acetyl-coenzyme A carboxyl transferase β chain (*accD6*), biotin carboxylase of acetyl-CoA carboxylase (*accA3*), biotin carboxyl carrier protein of acetyl-CoA carboxylase, 3-oxoacyl-(acyl-carrier-protein) synthase (*fabB*), trans-2-decenoyl-(acyl-carrier-protein) isomerase (*fabM*). | **FAS-II system:**  Malonyl CoA-acyl carrier protein transacylase (*fadD*), acyl carrier protein (*acpM*), long-chain-fatty-acid--CoA ligase (*fadD1*), acyl carrier protein phosphodiesterase (*acpH*), enoyl-(acyl-carrier-protein) reductase (*inhA*), acetyl-coenzyme A carboxyl transferase α chain (*accD6*), acetyl-coenzyme A carboxyl transferase β chain (*accD6*), biotin carboxylase of acetyl-CoA carboxylase (*accA3*), biotin carboxyl carrier protein of acetyl-CoA carboxylase. | **FAS-II system:**  Malonyl CoA-acyl carrier protein transacylase (*fadD*), long-chain-fatty-acid--CoA ligase (*fadD1*), acyl carrier protein (*acpM*), acyl carrier protein phosphodiesterase (*acpH*), enoyl-(acyl-carrier-protein) reductase (*inhA*), 3-hydroxyacyl-(acyl-carrier-protein) dehydratase (*hadA*), acetyl-coenzyme A carboxyl transferase α chain (*accD6*), acetyl-coenzyme A carboxyl transferase β chain (*accD6*), biotin carboxylase of acetyl-CoA carboxylase (*accA3*), biotin carboxyl carrier protein of acetyl-CoA carboxylase, 3-oxoacyl-(acyl-carrier-protein) synthase (*fabB*), trans-2-decenoyl-(acyl-carrier-protein) isomerase (*fabM*), β -ketoacyl synthase (*kasA*) |
| **Respiratory quinones** | Ubiquinone biosynthesis regulatory protein kinase (*ubiB*), ubiquinone biosynthesis protein (*ubiJ*), 3-demethylubiquinone-9 3-methyltransferase (*ubiG*), aromatic prenyltransferase 1 (*ubiA*),  UbiD family decarboxylase, flavin prenyltransferase (*ubiX*), 2-methoxy-6-polyprenyl-1,4-benzoquinol methylase (*ubiE*), chorismate--pyruvate lyase (*ubiC*), NADH-ubiquinone oxidoreductase, undecaprenyl-phosphate alpha-N-acetylglucosaminyl 1-phosphate transferase (*ubiH*), undecaprenyl diphosphate synthase (*ispB*), 2-polyprenyl-3-methyl-6-methoxy-1,4-benzoquinol hydroxylase (*ubiF*). | Ubiquinone biosynthesis regulatory protein kinase (*ubiB*), ubiquinone biosynthesis protein (*ubiJ*), 3-demethylubiquinone-9 3-methyltransferase (*ubiG*), aromatic prenyltransferase 1 (*ubiA*),  UbiD family decarboxylase, flavin prenyltransferase (*ubiX*), 2-methoxy-6-polyprenyl-1,4-benzoquinol methylase (*ubiE*), chorismate--pyruvate lyase (*ubiC*), NADH-ubiquinone oxidoreductase, undecaprenyl-phosphate alpha-N-acetylglucosaminyl 1-phosphate transferase (*ubiH*), undecaprenyl diphosphate synthase (*ispB*), 2-polyprenyl-3-methyl-6-methoxy-1,4-benzoquinol hydroxylase (*ubiF*). | Ubiquinone biosynthesis regulatory protein kinase (*ubiB*), ubiquinone biosynthesis protein (*ubiJ*), 3-demethylubiquinone-9 3-methyltransferase (*ubiG*), aromatic prenyltransferase 1 (*ubiA*), flavin prenyltransferase (*ubiX*), 2-methoxy-6-polyprenyl-1,4-benzoquinol methylase (*ubiE*), chorismate--pyruvate lyase (*ubiC*), NADH-ubiquinone oxidoreductase, undecaprenyl diphosphate synthase (*ispB*), undecaprenyl-phosphate alpha-N-acetylglucosaminyl 1-phosphate transferase (*ubiH*), 2-polyprenyl-3-methyl-6-methoxy-1,4-benzoquinol hydroxylase (*ubiF*). | Ubiquinone biosynthesis regulatory protein kinase (*ubiB*), ubiquinone biosynthesis protein (*ubiJ*), 3-demethylubiquinone-9 3-methyltransferase (*ubiG*), flavin prenyltransferase (*ubiX*), 2-methoxy-6-polyprenyl-1,4-benzoquinol methylase (*ubiE*), chorismate--pyruvate lyase (*ubiC*), NADH-ubiquinone oxidoreductase, undecaprenyl-phosphate alpha-N-acetylglucosaminyl 1-phosphate transferase (*ubiH*), ubiquinone biosynthesis hydroxylase, UbiH/UbiF/VisC/COQ6 family, monooxygenase, flavin-binding family (*ubiI*), decarboxylase family protein (*ubiD*), 4-hydroxybenzoate polyprenyltransferase (*ubiA*), undecaprenyl diphosphate synthase (*ispB*). |
| **Polar lipids** | Cardiolipin synthase (*clsA*, *clsB*, *clsC*), phosphatidylserine decarboxylase (*psd*), phosphatidyl glycerophosphatase A (*pgpA*), CDP-diacylglycerol--serine O-phosphatidyltransferase (*pssA*), phospholipase (*pld*). | Cardiolipin synthase (*clsA*, *clsB*, *clsC*), phosphatidylserine decarboxylase (*psd*), phosphatidyl glycerophosphatase A (*pgpA*), CDP-diacylglycerol--serine O-phosphatidyltransferase (*pssA*), phospholipase (*pld*). | Cardiolipin synthase (*clsA*, *clsB*, *clsC*), phosphatidylserine decarboxylase (*psd*), CDP-diacylglycerol--serine O-phosphatidyltransferase (*pssA*), phospholipase (*pld*), phosphatidylcholine synthase (*pcs*). | Cardiolipin synthase (*clsA*, *clsB*, *clsC*), phosphatidylserine decarboxylase (*psd*), phosphatidyl glycerophosphatase A (*pgpA*), CDP-diacylglycerol--serine O-phosphatidyltransferase (*pssA*), phospholipase (pld), phosphatidylcholine synthase (*pcs*). |

| **Supplementary Table 3.** Genes related to PGP properties. | | | |  |
| --- | --- | --- | --- | --- |
|  | ***Pseudomonas* sp. N4** | ***Pseudomonas* sp. N8^T^** | ***Pseudomonas* sp. L1^T^** | ***Pseudomonas* sp. SDT3^T^** |
| **IAA production** | - Indole-3-acetamide pathway:   Tryptophan 2-monooxygenase (*iaaM*).   - Indole-3-acetonitrile pathway:   Nitrilase.   - Indole-3-pyruvate pathway:   Aromatic-amino-acid aminotransferase.   - Tryptamine pathway:   Monoamine oxidase.   - Auxin efflux carrier. | - Indole-3-acetamide pathway:   Tryptophan 2-monooxygenase (*iaaM*).   - Indole-3-acetonitrile pathway:   Nitrilase.   - Indole-3-pyruvate pathway:   Aromatic-amino-acid aminotransferase.   - Tryptamine pathway:   Monoamine oxidase.   - Auxin efflux carrier. | - Indole-3-acetamide pathway:   Tryptophan 2-monooxygenase (*iaaM*).   - Indole-3-acetonitrile pathway:   Nitrilase, Nitrilase 2.   - Indole-3-pyruvate pathway:   Aromatic-amino-acid aminotransferase.   - Auxin efflux carrier. | - Indole-3-acetonitrile pathway:   Uncharacterized subgroup of the nitrilase superfamily.   - Indole-3-pyruvate pathway:   Aromatic-amino-acid aminotransferase.   - Tryptamine pathway:   Aromatic-L-amino-acid decarboxylase.  Monoamine oxidase   - Auxin efflux carrier. |
| **ACC deaminase activity** | 1-aminocyclopropane-1-carboxylate deaminase (*acdS*). | 1-aminocyclopropane-1-carboxylate deaminase (*acdS*). | – | – |
| **Phosphate solubilization** | Inorganic pyrophosphatase (*ppa*), NAD(P) transhydrogenase subunit alpha (*pntA*), NAD(P) transhydrogenase subunit beta (*pntB*), phosphatase (*nudJ*), alkaline phosphatase (*phoA, phoP*), phosphate-specific transport system accessory protein (*phoU*), phosphate regulon transcriptional regulatory protein (*phoB*), low-affinity inorganic phosphate transporter 1 (*pitAB*), polyphosphate kinase (*ppk*), exopolyphosphatase (*ppx*). | Inorganic pyrophosphatase (*ppa*), NAD(P) transhydrogenase subunit alpha (*pntA*), NAD(P) transhydrogenase subunit beta (*pntB*), phosphatase (*nudJ*), alkaline phosphatase (*phoA, phoP*), phosphate-specific transport system accessory protein (*phoU*), phosphate regulon transcriptional regulatory protein (*phoB*), low-affinity inorganic phosphate transporter 1 (*pitAB*), polyphosphate kinase (*ppk*), exopolyphosphatase (*ppx*). | Inorganic pyrophosphatase (*ppa*), acid phosphatase (*aphA*), alkaline phosphatase (*phoA*), sensor histidine kinase (*phoQ*), phosphate transport system regulatory protein (*phoU*), phosphate regulon transcriptional regulatory protein (*phoB*), alkaline phosphatase synthesis transcriptional regulatory protein (*phoP*), phosphate regulon sensor protein (*phoR*), exopolyphosphatase (*ppx*), phosphate ABC transporter permease protein (*pstAC*), phosphate ABC transporter ATP-binding protein (*pstB*), phosphate ABC transporter substrate-binding protein (*pstS*), inorganic triphosphatase, low-affinity inorganic phosphate transporter  (*phnA*), phosphonate ABC transporter ATP-binding protein (*phnCDE*). | Inorganic pyrophosphatase (*ppa*), acid phosphatase (*aphA*), NAD(P) transhydrogenase subunit alpha (*pntA*), NAD(P) transhydrogenase subunit beta (*pntB*), phosphate starvation inducible protein (*phoH*), phosphate-specific transport system accessory protein (*phoU*), phosphate regulon transcriptional regulatory protein (*phoB*), sensor histidine kinase (*phoQ*), phosphate regulon sensor protein (*phoR*), phosphate ABC transporter permease protein (*pstAC*), phosphate ABC transporter ATP-binding protein (*pstB*), phosphate ABC transporter substrate-binding protein (*pstS*), low-affinity inorganic phosphate transporter  (*phnA*), polyphosphate kinase (*ppk*), exopolyphosphatase (*ppx*), phosphohistidine phosphatase, (*sixA*). |
| **Siderophores production** | TonB-dependent ferric achromobactin receptor protein, iron siderophore sensor protein Sigma-70 factor FpvI, controling pyoverdin biosynthesis, iron siderophore receptor protein, ferrichrome receptor (*fiuA*),  Fe^3+^-pyochelin receptor (*fptA*), bacillibactin exporter (*ymfD*), enterobactin exporter (*entS*), ferric-anguibactin receptor (*fatA*). | TonB-dependent ferric achromobactin receptor protein, iron siderophore sensor protein Sigma-70 factor FpvI, controling pyoverdin biosynthesis, iron siderophore receptor protein, ferrichrome receptor (*fiuA*),  Fe^3+^-pyochelin receptor (*fptA*), bacillibactin exporter (*ymfD*), enterobactin exporter (*entS*), ferric-anguibactin receptor (*fatA*). | TonB-dependent ferric achromobactin receptor protein, acyl-homoserine lactone acylase (*pvdQ*), pyoverdine synthetase (*pvdF*), sigma-70 factor FpvI, Sigma factor PvdS-controling pyoverdin biosynthesis (*pvdS*), putative dipeptidase- pyoverdin biosynthesis (*pvdM*), pyoverdin biosynthesis protein (*pvdN*), pyoverdine responsive serine/threonine kinase (*pvdO*), pyoverdine ABC export system (*pvdE*), outer membrane ferripyoverdine receptor, non-ribosomal peptide synthetase modules, pyoverdine chromophore precursor synthetase (*pvdL*), pyoverdin biosynthesis protein (*pvdH*), siderophore biosynthesis non-ribosomal peptide synthetase modules (*PA2403_23*), putative thiamine pyrophosphate-requiring enzyme (*PA2403_24*), hypothetical protein in pyoverdin gene cluster (*PA2406*), cation ABC transporter, periplasmic cation-binding protein (*PA2407*), iron siderophore sensor protein, ferrichrome-iron receptor. | Ferric siderophore transport (*exbB*), TonB-dependent siderophore receptor protein. |
| **Biofilm formation** | Outer membrane secretin (*pgaA*), synthesis deacetylase (*pgaB*), synthesis N-glycosyltransferase (*pgaC*), synthesis auxiliary protein (*pgaD*), cyclic-di-GMP-binding biofilm dispersal mediator protein. | Outer membrane secretin (*pgaA*), synthesis deacetylase (*pgaB*), synthesis N-glycosyltransferase (*pgaC*), synthesis auxiliary protein (*pgaD*), cyclic-di-GMP-binding biofilm dispersal mediator protein. | Cyclic-di-GMP-binding biofilm dispersal mediator protein, pellicle/biofilm biosyntehsis inner membrane proteins (*pslACDEFGHIJKL*). | Cyclic-di-GMP-binding biofilm dispersal mediator protein, pellicle/biofilm biosyntehsis inner membrane proteins (*pslABCDEFG*). |
| **Motility** | Flagellar basal-body rod protein (*flgABCDFG*), flagellar hook protein (*flgE*), flagellar L-ring protein (*flgH*), flagellar P-ring protein (*flgI*), flagellar hook-associated protein (*flgKL*), flagellar biosynthesis proteins (*flhABF*, *flgN*, *FliOPQRS*), flagellar basal-body rod protein (*fliE*), flagellar M-ring protein (*fliF*), flagellar motor switch proteins (*fliGMN*), flagellar assembly protein (*fliH*), flagellum-specific ATP synthase (*fliI*), flagellar protein (*fliJ*), flagellar hook-basal body complex protein (*fliK*), sigma factor of RNA polymerase (*fliA*), flagellar motor rotation protein (*motAB*), falgellar sensor histidine kinase (*fleS*), flagellin protein (*flaA*), flagellar synthesis regulator (*fleN*), chemotaxis protein (*cheV*), RNA polymerase sigma-54 factor (*rpoN*), cell division protein (Peptidoglycan synthetase) (*ftsI*). | Flagellar basal-body rod protein (*flgABCDFG*), flagellar hook protein (*flgE*), flagellar L-ring protein (*flgH*), flagellar P-ring protein (*flgI*), flagellar hook-associated protein (*flgKL*), flagellar biosynthesis proteins (*flhABF*, *flgN*, *FliOPQRS*), flagellar basal-body rod protein (*fliE*), flagellar M-ring protein (*fliF*), flagellar motor switch proteins (*fliGMN*), flagellar assembly protein (*fliH*), flagellum-specific ATP synthase (*fliI*), flagellar protein (*fliJ*), flagellar hook-basal body complex protein (*fliK*), sigma factor of RNA polymerase (*fliA*), flagellar motor rotation protein (*motAB*), falgellar sensor histidine kinase (*fleS*), flagellin protein (*flaA*), flagellar synthesis regulator (*fleN*), chemotaxis protein (*cheV*), RNA polymerase sigma-54 factor (*rpoN*), cell division protein (Peptidoglycan synthetase) (*ftsI*). | Flagellar basal-body rod protein (*flgABCDFG, fliDE*), flagellar hook protein (*flgE*), flagellar hook-associated protein (*flgKL*), flagellar biosynthesis proteins (*flhABF*, *flgMN*, *FliLOPQRS*), flagellar M-ring protein (*fliF*), flagellar motor switch proteins (*fliGMN*), flagellar assembly protein (*fliH*), flagellum-specific ATP synthase (*fliI*), flagellar protein (*fliJ*), flagellar hook-basal body complex protein (*fliK*), sigma factor of RNA polymerase (*fliA*), flagellar motor rotation protein (*motAB*), sodium-type flagellar proteins (*motXY*), flagellin protein (*flaA*), flagellar synthesis regulator (*fleN*), chemotaxis protein (*cheV*), RNA polymerase sigma-54 factor (*rpoN*), cell division protein (Peptidoglycan synthetase) (*ftsI*), negative regulator of flagellin synthesis (Neg_reg). | Flagellar basal-body rod protein (*flgABCDFG, fliDE*), flagellar hook protein (*flgE*), flagellar L-ring protein (*flgH*), flagellar P-ring protein (*flgI*), flagellar hook-associated protein (*flgKL*), flagellar biosynthesis proteins (*flhABF*, *flgN*, *FliLOPQRS*), flagellar M-ring protein (*fliF*), flagellar motor switch proteins (*fliGMN*), flagellar assembly protein (*fliH*), flagellum-specific ATP synthase (*fliI*), flagellar protein (*fliJ*), flagellar hook-basal body complex protein (*fliK*), sigma factor of RNA polymerase (*fliA*), flagellar motor rotation protein (*motAB*), falgellar sensor histidine kinase (*fleS*), flagellin protein (*flaA*), chemotaxis protein (*cheV*), RNA polymerase sigma-54 factor (*rpoN*), cell division protein (Peptidoglycan synthetase) (*ftsI*). |
| –: Absence of gene in the genome. | | | | |


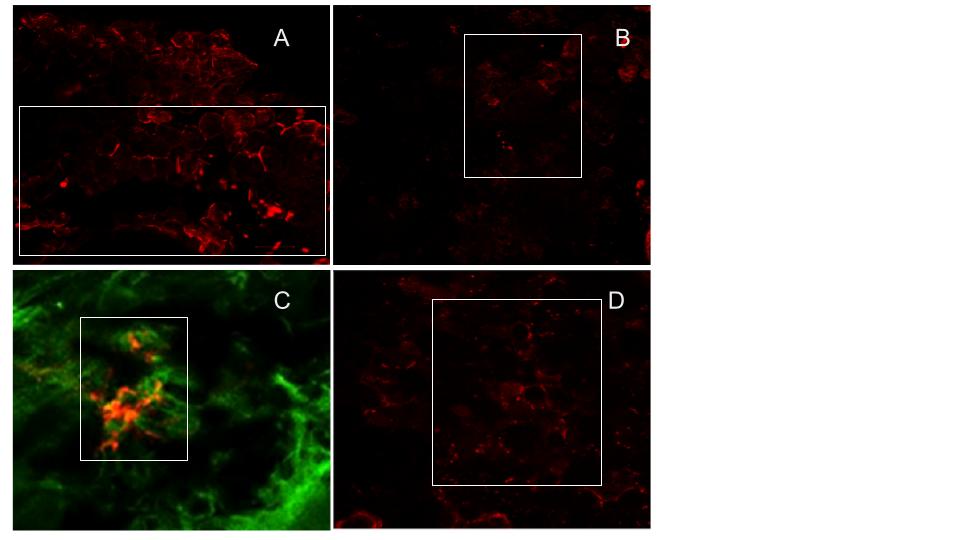


**Supplementary Fig. 2. Colonization of *Pseudomonas medicaginis* N8^T^ in the different legume species.**

Images of colonized nodules of co-inoculated plants with *Pseudomonas* *medicaginis* N8^T^ tagged with mCherry of lentil (A), pea (B), alfalfa (C), and bean (D) after 60 days sowing in pots with perlite and commercial substrate. White squares mark the nitrogen fixation zone. The images correspond to sections of approximately 0.5 mm of nodules taken with laser scanning confocal microscopy (Zeiss LSM 7 DUO, Zeiss, Jena, Germany) with a Plan-Apochromat 20×/0.8 M27 objective, 572–727 nm emission filters, and 561 nm (5.3%) excitation laser. Images were processed using ZEN2011 software (Zeiss, Jena, Germany).
